# The Effects of Cooking Process and Meat Inclusion on Pet Food Flavor and Texture Characteristics

**DOI:** 10.3390/ani4020254

**Published:** 2014-05-23

**Authors:** Kadri Koppel, Michael Gibson, Sajid Alavi, Greg Aldrich

**Affiliations:** 1 Sensory Analysis Center, Department of Human Nutrition, Kansas State University, 1310 Research Park Drive, Manhattan, KS 66502, USA; 2 Department of Grain Science and Industry, Kansas State University, Manhattan, KS 66506, USA; E-Mails: michael.gibson171@gmail.com (M.G.); salavi@ksu.edu (S.A.); aldrich4@ksu.edu (G.A.)

**Keywords:** aroma, baked, dog food, extruded, sensory analysis

## Abstract

**Simple Summary:**

The results of this research indicate that processing (baked *vs.* extruded) plays an important role in determining pet food product texture. In addition, raw ingredients (fresh meat *vs.* meal-based) did not consistently affect product sensory characteristics. These results may help pet food technologists better understand factors that affect palatability.

**Abstract:**

The pet food industry is an important portion of the food and feed industries in the US. The objectives of this study were (1) to determine cooking method (baking or extrusion), meat inclusion (0 or 20%), and extrusion thermal to mechanical energy ratios (low, medium, and high) effects on sensory and volatile properties of pet foods, and (2) to determine associations among sensory and volatile characteristics of baked and extruded pet foods. Descriptive sensory analysis and gas chromatography-mass spectrometry were used to analyze the pet food samples. It was found that baked samples were lighter in color (2.0–2.6 baked *vs.* 3.5–4.3 extruded, color intensity scale 0–15), and had lower levels of attributes that indicated rancidity (*i.e.*, fishy flavor; 0.3–0.6 baked, 0.6–1.5 extruded, scale 0–15), whereas extruded pet foods were more cohesive in mass, more friable, hard, and crisp, but less powdery than baked samples. Fresh meat inclusion tended to decrease bitterness and increase fishy flavor and cohesiveness of pet foods. High thermal to mechanical energy ratio during extrusion resulted in less musty and more porous kibbles. The main volatile compounds included aldehydes, such as hexanal and heptanal, ketones, and alcohols. Extruded samples did not contain methylpyrazine, while baked samples did not contain 2-butyl furan. Future studies should consider evaluating the relationship between sensory results and animal palatability for these types of foods.

## 1. Introduction

The pet food industry in the US continues to grow at a rate of approximately 4% per year, with a current estimated size of $19.7 billion [1]. Growth in this industry is driven by new product entries, such as naturally preserved diets; raw foods; “novel” ingredients like bison, potato, and peas; addition of nutraceuticals such as kelp, algae, acai berries; and an emphasis on fatty acid nutrition with foods containing fatty acids from fish and various seeds. While ingredients in pet foods are constantly changing, pet owners expect similar quality kibbles manufactured from any ingredient source. Most of the information in the pet food industry has been highly proprietary and, as a result, few publications have examined specific formulation or ingredient effects on pet food sensory properties.

Pet food processing has previously been evaluated from an extrusion perspective [2,3,4], and several studies have evaluated the effects of raw ingredients on pet food physical characteristics, palatability, and digestibility [5,6,7]. Other than extrusion, another popular processing technique used in dry pet food manufacturing is baking, mainly used in manufacturing pet treats. Gibson and Alavi described the effects of baking and extrusion on starch properties in pet foods [8]. However, to date, no studies have been found in which baked pet foods sensory characteristics have been characterized in comparison to extruded pet foods.

Sensory analysis of pet foods provides an additional tool in helping to understand both consumer and pet behavior. For example, Lin *et al*. studied selected processing effects such as fat source and moisture content with sensory analysis techniques [9]. These authors focused on selected pet food sensory properties such as aroma and appearance. Other studies have developed vocabularies to aid in a more thorough description of pet food sensory properties such as aroma, flavor, appearance, and texture. Examples are available on commercial dry dog food [10] and dry and canned cat foods [11,12]. Another study characterized volatile compounds in commercial pet foods and related those to sensory attributes [13]. Some associations, such as aldehydes and rancidity were detected among sensory qualities and various volatile molecules; however an approach that includes controlled ingredient composition and processing information would provide further insight.

The objectives of this study were (1) to determine processing, meat inclusion, and extrusion thermal and mechanical energy ratio level effects on sensory and volatile properties of pet foods, and (2) to determine associations among sensory and volatile characteristics of baked and extruded pet foods. The primary hypothesis being tested was that meat inclusion, processing method (baking *vs.* extrusion), and thermal to mechanical energy ratio during extrusion had an effect on pet food appearance, aroma, flavor, and texture properties. More specifically, it was hypothesized that baked pet food products would have a harder texture than extruded products, as the former are typically dense, while the latter are more expanded and porous in structure. Extensive scientific literature is available relating density to mechanical strength and hardness of porous or cellular products in general and extruded foods in particular [14,15]. It was also hypothesized that higher thermal to mechanical energy level ratios during extrusion would lead to harder products (due to reduced macromolecular degradation) and meat inclusion would result in dog food with less barnyard flavor. 

## 2. Experimental Section

### 2.1. Diet Formulation

Two maintenance dog food diets were formulated to be iso-nutritional based on carbohydrate, lipid, and protein content. Major variations within the diets were fresh meat inclusion (0 and 20%), chicken fat, and chicken by-product meal (Table 1). Dry ingredients were procured from Lortscher Agri Service, Inc. (Bern, KS, USA). Mechanically deboned chicken was acquired from C J Foods (Bern, KS, USA). Chicken fat was procured from American Dehydrated Foods (Springfield, MO, USA).

**Table 1:** Sample ingredients and nutritional composition.

Ingredients, %	0% Fresh Meat	20% Fresh Meat
Mechanically Deboned Chicken	0.00	20.00
Chicken Fat	5.32	2.34
Chicken By-Product Meal	20.94	10.91
Brewers Rice	21.21	18.84
Corn	21.21	18.84
Wheat	21.21	18.84
Beet Pulp	4.00	4.00
Corn Gluten Meal, 75%	3.00	3.00
Calcium Carbonate	0.75	0.75
Potassium Chloride	0.49	0.42
Sodium Chloride	0.46	0.43
Dicalcium Phosphate	0.87	1.12
Choline Chloride	0.20	0.20
Natural antioxidant, Dry	0.07	0.07
Natural antioxidant, Liquid	0.02	0.01
Trace Mineral Premix	0.10	0.10
Vitamin Premix	0.15	0.15
Nutritional Composition in the final product *		
Crude Protein	21.69–22.36	20.24–21.58
Crude Fat	5.42–8.86	5.35–-9.65
Ash	6.53–10.74	6.36–6.75
Crude Fiber	1.84–2.95	2.15–7.86
Moisture	4.20–6.67	4.23–6.25

* Nutritional Composition analyzed at University of Missouri Experiment Station Chemical Laboratories, w/w%.

### 2.2. Grinding and Mixing

Whole grains (corn and wheat) were ground using a Fitz mill (Model D, Fitzpatrick Company, Elmhurst, IL, USA) to pass through a 1532-0040 screen with a round hole opening of 1.02 mm. Dry ingredients (Table 1) were mixed together in a double-ribbon horizontal mixer (Wenger Manufacturing, Sabetha, KS, USA). Major ingredients (brewer’s rice, corn, wheat, beet pulp, chicken by-product meal, and corn gluten meal) were mixed for three minutes, and then minor ingredients (calcium carbonate, potassium chloride, sodium chloride, dicalcium phosphate, choline chloride, dry antioxidant, trace mineral and vitamin premixes) were added into the mixer for an additional two minutes. Post mixing, the entire batch was ground through the same milling system in order to achieve a more uniform particle size for the entirety of dry ingredients (major and minor ingredients).

### 2.3. Processing

Two cooking methods—extrusion or baking—were used to manufacture the pet food samples. For extruded samples three thermal energy input levels (Low; LE, Medium; ME, and High; HE) and 0 or 20% meat inclusion were used resulting in six pet food treatments: 0LE, 20LE, 0ME, 20ME, 0HE, and 20HE. For baked samples one processing time (11 min) was used with 0 and 20% meat inclusion resulting in two baked samples (0B and 20B). The extrusion and baking processes are described in detail below.

### 2.4. Extrusion

**Figure 1:**
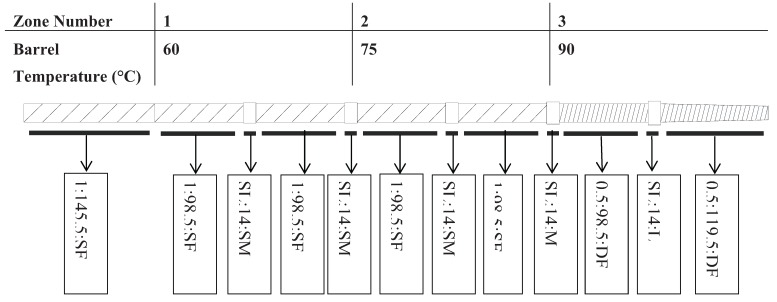
Schematic showing pilot scale single screw extruder profile and barrel temperature setting. The screw element codes a:b:x imply the following: a = 1 (full pitch screw), 0.5 (half pitch screw), SL (steam lock element); b = element length (mm); x = SF (single flighted screw), DF (double flighted screw), SM (small diameter steam lock), M (medium diameter steam lock), L (large diameter steam lock). The last screw element is a conical shaped segment.

Diets were processed on a single screw extruder (X-20, Wenger Manufacturing, Sabetha, KS, USA) with the screw profile displayed in Figure 1. Processing conditions, e.g., extruder screw RPM (350 425 RPM as Medium, and 500 RPM) and preconditioner steam input (8 kg/h, 12 kg/h, and 16 kg/h as High) were varied to achieve different thermal to mechanical ratios. The specific ratios used were 500 RPM and 8 kg/h steam input for Low thermal to mechanical energy ratio, 425 RPM and 12 kg/h steam input for Medium, and 350 RPM and 16 kg/h steam input for High thermal to mechanical energy ratios.

The extruder screw profile, starting at the feed throat, was a single flight single pitch screw and tapered down to a double flight half pitch screw element. Shear locks increased in size, from small to large, in between each screw element after the inlet screw element. The extruder screw profile was selected to convey and compress material as the pet food mash passed through the extruder. 

The natural liquid antioxidant was mixed into chicken fat prior to being pumped into the middle section of the preconditioner using a Seepex pump (Range, MO, USA). Mechanically deboned chicken was pumped into the final zone of the preconditioner using a Waukesha Sanitary pump (Delavan, WI, USA). Each pump was calibrated to the required set points for each formulation prior to extrusion.

Processing moistures were calculated prior to extrusion to achieve 27% in-barrel moisture. The introduction of fresh meat and oil changed flow rates of the dry feed rate and moisture additions. 

After extrusion, kibbles were pneumatically conveyed to a double pass dryer and single pass cooler (4800 series, Wenger Manufacturing, Sabetha, KS, USA). Dryer settings were 104 °C for 10 min per pass, followed by cooling with room temperature air for 10 minutes. The dryer and cooler were both continuous, perforated belt type, and were part of an integrated system. Samples were collected at the end of the cooler and stored in hermetically sealed bags at ambient temperature. All samples were stored for similar periods of time until further testing.

### 2.5. Baking

The baked treatments were mixed together in a planetary mixer (HL800 Hobart, Troy, OH, USA) at the American Institute of Baking (Manhattan, KS, USA). The mixes were comprised of the dry mix described above, mechanically deboned chicken, chicken fat, and water. The final dough moisture levels were targeted to be 33–34% on a wet basis (w.b.). The dough was passed through a rotary molder (RM14B81, Weidenmiller Company, Itasca, IL, USA) to make frustum-shaped kibbles. 

The rotary molded kibbles were placed into a rack oven (Model 626, Revent, Inc., Somerset, NJ, USA) preheated to 220 °C and baked for 11 min. Post baking, kibbles were placed in a drying oven at 50 °C for 5 h to drive off excess moisture. To confirm the dog food achieved the final target moisture level below 10% w.b., moisture content was measured following AOAC method 930.15.

### 2.6. Descriptive Sensory Analysis

#### 2.6.1. Panelists

Five highly trained panelists from the Sensory Analysis Center, Kansas State University (Manhattan, KS, USA) participated in this study. All of the panelists had completed 120 h of general descriptive analysis training with a variety of food products. The training included techniques and practice in attribute identification, terminology development, and intensity scoring. Each of the panelists had more than 1,000 h of testing experience with a variety of food products. For this project, the panelists received further orientation on dried dog food using samples that may or may not be included in the study. Panels of similar size and training have been reported in other recent research [16,17,18,19].

#### 2.6.2. Sample Presentation and Evaluation

Each sample was served in a ~100 mL plastic cup for appearance, texture, and flavor evaluation, and in a medium snifter covered with a watch glass for the evaluation of aroma. The amount of product in the snifter was 3 g. Samples were prepared 30 min prior to the testing and were coded with three-digit random numbers. All of the samples were evaluated in a randomized order in duplicate for appearance, texture, flavor, and aroma using attributes selected from a lexicon developed for this product category by Di Donfrancesco *et al*. [10]. Barnyard, brothy, toasted, brown, grain, vitamin, stale, meaty, musty, oxidized oil, cardboard, liver, and fish aroma, flavor, and aftertaste attributes were evaluated in all samples. In addition sour, salty, sweet, and bitter taste and aftertaste and metallic aftertaste attributes were evaluated. Brown color intensity, porous, grainy, and fibrous appearance characteristics and cohesiveness of mass, fracturability, hardness, powdery, crispness, and mouth-coating texture attributes (powdery and mouthcoat) were evaluated. Six 2-hour sessions were held for the evaluation phase. For the evaluation, a numeric scale of 0–15 with 0.5 increments where 0 represents none and 15 extremely high was applied to each attribute to provide a measure of intensity. Each panelist individually assigned intensities to the attributes present in the sample according to their perception of the appearance, aroma, flavor, and texture references included in the lexicon. The panelists were provided with a definition sheet with the list of attributes and their definitions as well as reference materials for each attribute according to Di Donfrancesco *et al*. [10]. 

The panelists were asked to chew one kibble for flavor and texture evaluation. The panelists were instructed to expectorate samples after evaluation. Panelists were provided with apple slices, unsalted crackers, purified water, and toothbrushes for palate cleansing in between the evaluations. The testing room was at 21 ± 1 °C and 55 ± 5% relative humidity.

### 2.7. Volatile Compounds Measurement

#### 2.7.1. Extraction Procedure of Volatile Aroma Compounds

The extraction method chosen for studying the aroma profile in the dry dog foods was headspace-solid phase microextraction (HS-SPME) as described by Koppel *et al*. [13]. The samples were ground in pestle & mortar, then a 0.5 g samples was weighed into a 10 mL screw-cap vial with a polytetrafluoroethylene/silicone septum. Exactly 0.48 mL distilled water was added to the ground sample in the vial. To this an internal standard consisting of 0.02 mL 1,3-dichlorobenzene (98%, Sigma Aldrich, St. Louis, MO, USA) dissolved in hexane (mixture of isomers, optima grade, Fisher Scientific; Pittsburgh, PA, USA), with final concentration in the sample of 0.2 mg/kg was added. The vials were equilibrated for 10 min at 40 °C in the autosampler (Pal system, model CombiPal, CTC Analytics, Zwingen, Switzerland) and agitated at 250 rpm. After the equilibration, a 50/30 µm divinylbenzene/carboxen/polydimethylsiloxane fiber was exposed to the sample headspace for 30 min at 40 °C. The fiber method was chosen for its high capacity of trapping volatile compounds in food products [20]. 

After sampling, the analytes were desorbed from the SPME fiber coating prior in the GC injection port at 270 °C for 3 min in splitless mode.

#### 2.7.2. Chromatographic Analyses

The isolation, tentative identification, and semi-quantification of the volatile compounds were performed on a gas chromatograph (Varian GC CP3800; Varian Inc., Walnut Creek, CA, USA), coupled with a Varian mass spectrometer (MS) detector (Saturn 2000). The GC-MS system was equipped with an RTX-5MS (Crossbond^®^ 5% diphenyl/95% dimethyl polysiloxane) column (Restek, U.S., Bellefonte, PA, USA; 30 m × 0.25 mm × 0.25 µm film thickness). The initial temperature of the column was 40 °C held for 4 min; the temperature was then increased by 5 °C per min to 260 °C, and held at this temperature for 7 min. All samples were analyzed in triplicates. The quantities of volatile compounds were calculated against the internal standard peaks.

Most of the compounds were identified using two different analytical methods: (1) mass spectra (>80%) and (2) Kovats indices (NIST/EPA/NIH Mass Spectral Library, Version 2.0, 2005). Identification was considered tentative when it was based on only mass spectral data. The retention times for a C7–C40 saturated alkane mix (Supelco Analytical, Bellefonte, PA, USA) was used to determine experimental Kovats indices for the volatile compounds detected.

### 2.8. Data Analysis

Significant treatment effects (P < 0.05) were determined for meat inclusion (all samples), processing (all samples), and thermal input level (extruded samples only) using SAS Glimmix procedure (Version 9.3, SAS Institute Inc., Cary, NC, USA).

Partial least squares regression (PLSR) study is a multivariate statistical technique that has been used by several researchers for creating external preference maps for determining relationships between descriptive data (X-matrix) and consumer acceptability data (Y-matrix) [21]. PLSR can be used to correlate the instrumental volatile data (X-matrix) and descriptive sensory data (Y-matrix), an approach similar to the one reported by Koppel *et al*. [13]. This procedure using sensory flavor attributes data as the Y-matrix and volatile compounds data as the X-matrix was conducted in our study using Unscrambler (version 10.2; Camo Software; Oslo, Norway).

## 3. Results and Discussion

### 3.1. Descriptive Sensory Analysis

Most aroma, flavor, and aftertaste attributes were scored at low intensity levels (0–5 on a scale from 0 to 15; Table 2(a,b)). According to Di Donfrancesco *et al*. most commercial dry dog food samples exhibit low flavor intensities [10]. The same authors reported meaty flavor in dry dog foods to be a complex attribute usually detected at low levels. In our study, meaty flavor was not identified by the panelists, although it was included in the attribute list. This indicates that meaty flavor probably results from a surface flavoring applied to commercial products that was not included in our formulas. A similar situation was observed for sweet taste; wherein, sweetness in dry dog foods may result from added dextrose, cane syrup or other flavoring agents such as sweet-tasting amino acids to enhance overall sweetness.

Fresh meat inclusion tended to affect pet food bitterness (P = 0.03) and fish flavor (P = 0.008; Table 2a). Pet foods manufactured with fresh meat tended to be less bitter but higher in fish flavor than samples manufactured without fresh meat. The fish flavor may be related to rancid fatty acid flavor notes. Rancidity in foods, including pet foods, is recognized by volatile compounds that result from the oxidation of fatty acids (e.g., hexanal, heptanal, octanal). In pet foods manufactured with animal by-product meals these volatiles are likely to be present at a lower level because the rendering process includes cooking the materials at 115–145 °C [22]. Fresh meat inclusion was also observed to increase cohesiveness of mass characteristics in pet food samples. This is probably related to the enhanced binding properties of meat fibers in the fresh meat treatment. 

Thermal to mechanical energy ratio had an effect on sensory characteristics among the extruded pet food samples. A higher ratio tended to decrease brown color intensity and increase porous, grainy, and fibrous appearance; these results are in line with findings by Cheng *et al*. [23]. Musty flavor was more pronounced in pet food samples manufactured at lower thermal input. In addition some texture characteristics, such as fracturability, hardness, and crispness were affected by thermal input. Fracturability (defined as “the force with which the sample ruptures”) was highest for medium thermal input samples, while hardness (defined as “the force required to bite completely through the sample with molar teeth”) was scored at highest intensity for low thermal samples. Initial crispness, defined as “the intensity of audible noise at first chew with molars” was scored as more intense for medium and high thermal input samples. This is consistent with the hypothesis stated earlier that increased thermal energy as compared to mechanical energy input leads to lesser macromolecular degradation and a product with a stronger solid matrix. 

Baking is a process that involves only thermal energy (heat), while extrusion involves both thermal and mechanical energy (friction) under pressure [24]. Intuitively one would expect the texture characteristics of baked and extruded products to be different. This was substantiated by our study. Cooking process (baked *vs.* extruded) effects were different for all sensory attributes shown in Table 2(a,b) except for grainy appearance and musty flavor. Baked pet food samples resulted in a more porous appearance and were lower in brown color intensity. According to Cowell *et al*. baking would result in more uniform products when compared to extrusion, and this may have an influence on consumer perception [25]. In addition, baked pet foods were found to be lower in almost all flavor attributes than extruded samples except for bitter taste and aftertaste. Furthermore baked samples were lower in cohesiveness of mass (P = 0.0003), hardness (P < 0.0001), and initial crispness (P < 0.0001), but more intense in powdery (P < 0.0001) and mouthcoat attributes (P < 0.0001). The lower hardness of baked products was contrary to the hypothesis stated earlier that these higher density products would have a greater hardness. The primary reason was the limited gelatinization of starch in the baked products due to absence of mechanical energy input as compared to almost complete gelatinization in extruded products [8]. Lower starch gelatinization would lead to a lesser binding of various components in the formulation and weaker solid matrix, which in turn would result in reduced overall product hardness. According to Houpt and Smith, dogs have certain preferences when it comes to food flavor and texture, such as moist and canned food is preferred over dry, and beef may be preferred over pork, chicken, and other meats [26]. No information was found in the scientific literature on baked *vs.* extruded texture preferences, and this presents an opportunity for future research.

**Table 2a:** Descriptive sensory analysis attributes (mean values) for pet foods differing in cooking technique, extrusion thermal input, and amount of fresh meat included in the formula reported on an intensity scale of 0 to 15 with 1 being none to 15 extremely intense.

Attribute	Sample									Fresh Meat effect	TI effect	Proc. effect
	0B	20B	0LE	20LE	0ME	20ME	0HE	20HE	STDEV			
Brown ap	2.7	2.9	4.3	3.9	4.0	3.7	3.6	3.5	0.860	NS	0.003	<0.0001
Porous ap	3.9	3.3	1.9	1.8	2.1	2.2	3.2	3.1	1.136	NS	<0.0001	<0.0001
Grainy ap	2.4	2.3	2.2	2.3	2.6	2.3	2.9	2.7	0.776	NS	0.004	NS
Fibrous ap	0.8	1.0	1.5	1.0	1.7	1.5	1.7	1.9	0.839	NS	0.03	0.0007
Toasted ar	1.4	1.6	1.6	1.6	1.7	1.8	1.7	1.8	0.467	NS	NS	0.018
Brown ar	0.5	0.5	0.3	0.3	0.1	0.5	0.2	0.3	0.597	NS	NS	0.02
Stale ar	1.5	1.3	1.6	1.9	1.9	1.9	1.6	1.6	0.746	NS	NS	0.018
Fish ar	0.0	0.3	0.4	0.6	0.3	0.5	0.5	0.5	0.622	NS	NS	0.017
Toasted fl	1.4	1.6	1.9	1.8	1.7	1.7	1.9	2.1	0.509	NS	NS	0.004
Grain fl	2.9	3.2	3.3	3.3	3.5	3.5	3.4	3.3	0.665	NS	NS	0.01
Vitamin fl	0.4	0.7	1.1	0.8	1.0	0.6	0.8	0.8	0.689	NS	NS	0.03
Stale fl	2.1	2.0	2.2	2.4	2.2	2.1	2.4	2.4	0.774	NS	NS	0.02
Bitter	4.6	4.2	4.3	4.2	4.3	3.9	4.0	3.9	0.740	0.03	NS	0.03
Musty fl	1.3	1.2	1.6	1.2	1.1	1.1	1.0	1.0	0.890	NS	0.03	NS
Ox oil fl	2.6	2.6	2.7	3.0	3.0	2.8	3.0	2.8	0.625	NS	NS	0.007
Fish fl	0.3	0.7	1.0	1.2	1.0	1.1	0.6	1.5	0.938	0.008	NS	0.001

TI: thermal:mechanical energy ratio; L: Low; M: Medium; H: High; B: baked; E: Extruded; Proc.: processing; Ox. Oil: oxidized oil; ap: appearance; ar: aroma; fl: flavor; STDEV: standard deviation.

**Table 2b:** Descriptive sensory analysis attributes mean values, formulation and processing effects.

Attribute	Sample									Fresh Meat effect	TI effect	Proc effect
	0B	20B	0LE	20LE	0ME	20ME	0HE	20HE	STDEV			
Barn. at	2.7	2.5	2.9	2.9	3.1	2.8	2.8	2.7	0.711	NS	NS	0.03
Salty at	1.3	1.2	1.7	2.0	1.9	1.6	1.5	1.8	0.827	NS	NS	0.004
Bitter at	4.8	4.5	4.5	4.5	4.3	4.5	4.1	4.1	0.896	NS	NS	0.04
Vitamin at	0.1	0.4	1.1	0.5	1.0	0.4	0.5	0.7	0.707	NS	NS	0.003
Musty at	1.2	1.2	0.8	0.9	0.8	1.2	0.7	0.8	0.992	NS	NS	0.005
Ox oil at	2.4	2.2	2.5	2.5	2.6	2.5	2.5	2.8	0.735	NS	NS	0.01
Liver at	0.7	0.9	1.5	1.3	1.5	1.4	1.2	1.5	1.052	NS	NS	0.0003
Fish at	0.2	0.4	1.0	0.8	0.9	1.0	0.8	1.0	0.799	NS	NS	0.0004
Coh. mass	2.5	3.0	3.6	3.8	3.7	4.9	3.4	4.3	1.552	0.01	NS	0.0003
Fractu-rability	5.3	5.0	6.9	6.8	7.9	8.0	7.7	7.3	2.087	NS	0.048	<0.0001
Hardness	5.1	5.1	8.5	8.3	8.0	8.0	7.9	7.5	1.791	NS	0.001	<0.0001
Powdery	3.7	3.8	2.3	2.5	2.5	2.4	2.1	2.3	1.111	NS	NS	<0.0001
Crispness	6.0	5.9	9.9	9.8	10.7	10.7	10.7	10.5	2.409	NS	0.04	<0.0001
Mouthcoat	2.9	2.4	2.1	2.2	1.8	2.1	2.0	1.9	0.581	NS	NS	<0.0001

TI: thermal:mechanical energy ratio; L: Low; M: Medium; H: High; B: baked; E: Extruded; Proc.: processing; Ox. Oil: oxidized oil; Barn: barnyard; at: aftertaste; STDEV: standard deviation.

**Table 3a:** Volatile compounds in samples (μg/kg).

No	Volatile	KI Exp	KI Lit	Sample							
				0B	20B	0LE	20LE	0ME	20ME	0HE	20HE
	Alcohols										
A1	Octen-3-ol	960	961d	1.61±0.70	1.80±0.47	3.15±0.74	2.69±0.57	3.07±0.43	3.62±0.26	2.17±0.28	2.30±0.37
A2	2-Decen-1-ol	1187	*1101c*	ND	ND	0.32±0.02	0.32±0.10	0.30±0.00	0.45±0.02	0.20±0.02	0.32±0.04
A3	2-Butyl octanol	1298	*1201c*	2.44±0.71	3.34±0.94	0.76±0.05	2.63±0.38	2.52±0.29	2.94±0.35	0.85±0.07	2.13±0.13
	**Total alcohols**			**4.06**	**5.15**	**4.23**	**5.64**	**5.89**	**7.02**	**3.22**	**4.75**
	Aldehydes										
A4	3-Methylbutanal	NA	654c	1.41±0.82	1.74±0.30	1.06±0.04	0.70±0.28	1.28±0.23	0.99±0.15	1.13±0.30	0.89±0.14
A5	Hexanal	NA	800a	21.34±11.53	23.89±5.24	54.47±7.34	67.03±20.77	50.21±6.98	85.77±13.68	39.06±5.91	54.46±6.52
A6	3-Furaldehyde	NA	829b	0.50±0.30	0.55±0.23	0.34±0.01	0.36±0.14	0.38±0.03	0.40±0.05	0.45±0.05	0.40±0.08
A7	2-Hexenal(E)	NA	854b	0.30±0.13	0.31±0.08	0.35±0.02	0.45±0.05	0.33±0.03	0.48±0.07	0.24±0.03	0.31±0.03
A8	Heptanal	872	872d	1.96±0.89	2.24±0.49	3.90±0.70	3.46±1.17	4.07±0.63	4.95±0.73	3.21±0.43	3.63±0.58
A9	3-Methylthiopropanal	NA	902c	0.31±0.18	0.49±0.14	0.25±0.05	0.16±0.02	0.26±0.03	0.22±0.04	0.26±0.02	0.20±0.04
A10	2-Heptenal(Z)	902	904d	1.31±0.66	1.77±0.64	1.65±0.29	1.81±0.19	1.54±0.21	2.50±0.11	0.90±0.03	1.21±0.10
A11	Benzaldehyde	909	910d	4.56±2.62	4.30±1.06	6.34±1.10	5.36±2.28	6.42±1.02	6.08±0.90	5.16±0.93	4.98±1.18
A12	Octanal	976	977d	1.09±0.47	1.22±0.28	3.05±0.57	3.16±1.00	3.02±0.25	4.55±0.61	2.52±0.36	3.34±0.51
A13	2-Ethyl-2-hexenal	979	NA	ND	ND	0.29±0.05	0.12±0.03	0.38±0.05	0.25±0.04	0.33±0.02	0.25±0.07
A14	Benzeneacetaldehyde	1006	1006d	1.24±0.52	1.59±0.39	1.31±0.10	1.02±0.02	1.56±0.23	1.57±0.39	1.58±0.02	1.00±0.06
A15	2-Octenal	1010	1010d	1.20±0.35	1.36±0.31	1.81±0.10	2.04±0.22	1.03±0.05	1.47±0.20	1.56±0.05	0.95±0.14
A16	Nonanal	1082	1082d	1.77±0.64	2.03±0.41	4.22±0.62	5.21±1.66	17.47±3.62	6.13±0.59	3.46±0.45	4.95±0.77
A17	2-Nonenal	1119	1142c	0.34±0.12	0.38±0.11	0.39±0.03	0.54±0.07	0.36±0.05	0.52±0.07	ND	0.45±0.02
A18	2-Butyl-2-octenal	1366	1366d	0.15±0.02	0.09±0.02	0.22±0.04	0.23±0.11	0.23±0.09	0.13±0.04	0.22±0.09	0.14±0.01
	**Total aldehydes**			**37.49**	**41.96**	**79.66**	**91.65**	**88.54**	**115.97**	**60.09**	**77.16**
	Ketones										
A19	2-Heptanone	864	865d	1.24±0.65	1.26±0.31	1.23±0.16	0.90±0.25	1.56±0.03	1.15±0.04	1.34±0.30	1.24±0.24
A20	2,5-Octanedione	962	983c	1.16±0.63	1.83±0.46	3.32±0.42	4.59±0.97	2.41±0.21	5.19±0.77	2.02±0.18	3.63±0.24
A21	6-Methyl, 5-hepten-2-one	963	965d	0.31±0.17	0.26±0.02	0.86±0.22	0.65±0.15	0.76±0.12	0.87±0.07	0.46±0.13	0.77±0.10
A22	3-Octen-2-one	998	999d	ND	ND	0.75±0.07	1.24±0.30	0.72±0.10	2.49±0.38	0.83±0.03	1.73±0.11
A23	2-Nonanone	1071	1072d	0.08±0.03	0.07±0.02	0.40±0.03	0.30±0.08	0.54±0.02	0.50±0.06	0.57±0.04	0.45±0.02
	**Total ketones**			**2.79**	**3.42**	**6.56**	**7.68**	**5.99**	**10.20**	**5.24**	**7.81**

ND: not detected; NA: not available; a: [27]; b: [28]; c: [29]; d: [13]; italic: different column; KI exp: Kovacs Indice calculated experimentally; KI lit: Kovacs Indice from literature.

**Table 3b:** Volatile compounds in samples (μg/kg).

No	Volatile	KI Exp	KI Lit	Sample							
				0B	20B	0LE	20LE	0ME	20ME	0HE	20HE
	Esters										
A24	Methyl butyrate	NA	724c	0.30±0.13	0.22±0.06	0.16±0.05	0.17±0.07	0.16±0.03	0.16±0.05	0.16±0.03	0.14±0.06
A25	Methyl hexanoate	883	*916c*	0.33±0.20	0.45±0.17	0.43±0.18	0.50±0.25	0.40±0.10	0.55±0.16	0.32±0.08	0.42±0.10
A26	Methyl octanoate	1092	*1107c*	0.03±0.02	0.06±0.03	ND	ND	ND	ND	ND	ND
A27	Propanoic acid 2-methyl butylester	1142	NA	3.70±0.37	2.93±1.20	1.50±1.26	2.96±2.72	1.10±0.58	2.22±2.39	2.04±1.31	1.53±1.10
	**Total esters**			**4.36**	**3.66**	**2.09**	**3.62**	**1.66**	**2.94**	**2.52**	**2.09**
	Pyrazines										
A28	Methylpyrazine	NA	828b	0.28±0.17	0.39±0.20	ND	ND	ND	ND	ND	ND
A29	Pyrazine, 2,5-dimethyl	NA	911c	0.34±0.14	0.39±0.09	0.26±0.05	0.18±0.04	0.29±0.03	0.23±0.04	0.19±0.02	0.14±0.02
	**Total pyrazines**			**0.62**	**0.78**	**0.26**	**0.18**	**0.29**	**0.23**	**0.19**	**0.14**
	Furans										
A30	2-Butylfuran	893	895d	ND	ND	0.25±0.04	0.34±0.08	0.28±0.03	0.28±0.05	0.27±0.11	0.39±0.10
A31	2-Pentylfuran	993	994d	1.00±0.38	1.01±0.14	4.81±0.56	3.90±1.58	5.87±1.19	4.98±0.92	7.14±1.19	6.00±0.82
	**Total furans**			**1.00**	**1.01**	**5.07**	**4.24**	**6.15**	**5.26**	**7.42**	**6.38**
	Acids										
A32	4-Methyl pentanoic acid	770	NA	0.35±0.24	0.36±0.20	0.69±0.14	0.37±0.17	0.73±0.26	0.47±0.20	0.40±0.08	0.30±0.01
	Terpenes										
A33	1-R-α-pinene	929	932d	ND	ND	0.25±0.01	0.16±0.05	0.34±0.04	0.28±0.04	0.30±0.06	0.26±0.03
A34	Limonene (L)	1040	1041d	0.08±0.06	0.43±0.17	0.56±0.04	0.34±0.08	1.12±0.19	0.94±0.22	0.77±0.07	0.78±0.07
	**Total terpenes**			**0.08**	**0.43**	**0.81**	**0.50**	**1.46**	**1.22**	**1.08**	**1.04**
	Alkenes										
A35	3-Dodecene(E)	914	NA	1.51±0.22	1.43±0.20	1.99±0.16	1.84±0.08	1.91±017	2.23±0.24	2.10±0.22	1.76±0.39
	Other compounds										
A36	3-Hydroxytoluene	953	NA	ND	ND	2.68±0.47	2.48±0.65	3.15±0.26	2.16±0.41	3.15±0.22	3.12±0.39
A37	Indole	1120	1136c	0.23±0.09	0.14±0.04	0.33±0.03	0.22±0.05	0.39±0.02	0.33±0.01	0.34±0.04	0.29±0.03
	**Total other compounds**			**0.23**	**0.14**	**3.01**	**2.70**	**3.54**	**2.48**	**3.49**	**3.41**
	**Total volatiles**			**52.50**	**58.34**	**104.36**	**118.42**	**116.16**	**148.02**	**85.73**	**104.84**

ND: not detected; NA: not available; a: [27]; b: [28]; c: [29]; d: [13]; italic: different column; KI exp: Kovacs Indice calculated experimentally; KI lit: Kovacs Indice from literature.

### 3.2. Volatile Compounds

Overall 37 volatile compounds were found in the pet food samples (Table 3(a,b)). These volatiles included alcohols (three compounds), aldehydes (15 compounds), ketones (five compounds), esters (four compounds), pyrazines (two compounds), furans (two compounds), alkenes (one compound), acids (one compound), and terpenes (two compounds). Koppel *et al*. looked at volatiles in commercial dry dog food samples and identified 54 volatile compounds [13]. Those samples were manufactured with a variety of grains, proteins, and added flavorings that likely contributed to the greater number of volatiles detected than we found in this study.

The cooking process and meat level in the formulation seemed to have an effect on the volatiles content. Total concentration of volatiles was higher in the extruded samples (85–148 µg/kg) when compared to the baked samples (52–58 µg/kg). In addition, the meat-added samples seemed to be more aromatic (58–148 µg/kg) when compared to the no meat-added samples (52–116 µg/kg). Overall the most aromatic sample was the meat-added medium thermal input extruded pet food.

Some compounds were found only in baked or extruded samples. For example 2-decen-1-ol, 2-ethyl-2-hexenal, 3-octen-2-one, 2-butylfuran, 3-hydroxytoluene, and 1-R-α-pinene (tentative identification) were present in extruded samples, but were not detected in baked foods. Several of these compounds are probably odor-active, although the concentrations found in extruded pet foods were low. According to Eyres *et al*. 2-decen-1-ol could have wet dog aromatics [30]; 1-R-α-pinene may have musty and pungent characteristics (Pherobase); 3-octen-2-one was found in poultry byproduct meal and was claimed to contribute to the fatty, hay, and cardboard-like aromatics [31]. Methylpyrazine and methyl octanoate were present in baked foods, but were not detected in extruded samples, and thus may be processing related in their formation. Methylpyrazine may carry popcorn aromatics [29]. The aromatics that these compounds may have could be further studied using a gas-chromatograph olfactometer (GC-O).

According to Mottram, aldehydes are common lipid oxidation products [32]. Pet food samples in this study contained chicken fat, chicken meat, and chicken byproduct meal. Aldehydes were the most abundant group of volatiles identified, with concentrations ranging from 37–115 µg/kg, and hexanal was found at the highest concentration among aldehydes (21–85 µg/kg). Hexanal is known to be associated with rancid, fatty, and oxidized oil aromatics [13]. According to Greenberg poultry byproduct meal lacks the necessary volatiles to give the meal savory and pleasant meat odor characteristics [31]. In pet foods this can be turned around using meat-like flavorings and coatings.

### 3.3. Associations between Sensory Attributes and Volatile Compounds

**Figure 2:**
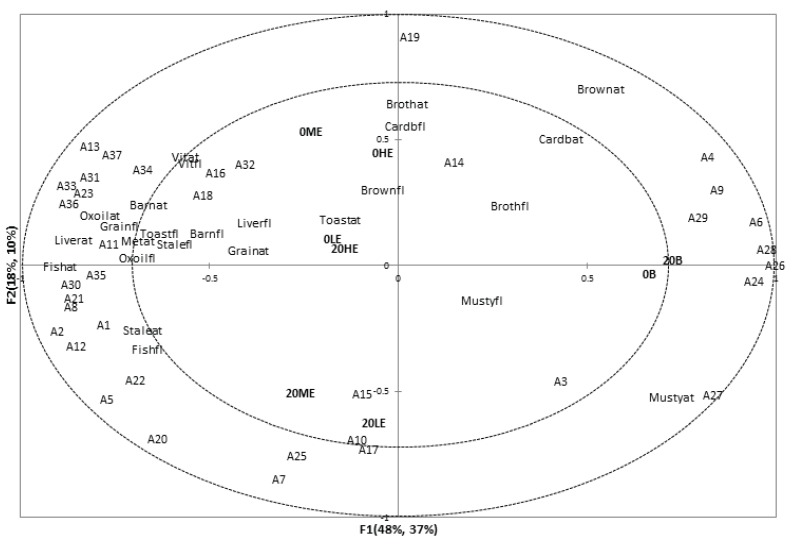
Partial Least Squares Regression Factors 1 and 2. Suffixes: ap – appearance; ar – aroma; fl – flavor; at – aftertaste.

**Figure 3:**
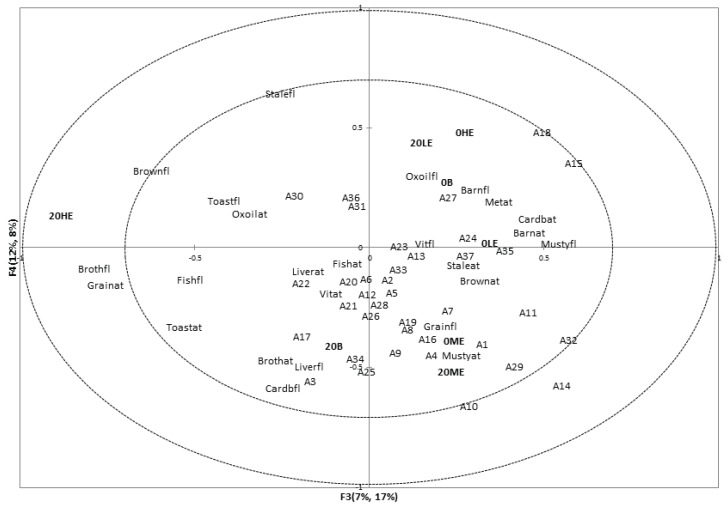
Partial Least Squares Regression Factors 3 and 4. Suffixes: ap – appearance; ar – aroma; fl – flavor; at – aftertaste.

Partial Least Squares Regression (PLS-R) maps (Figure 2 and Figure 3) show associations between sensory flavor attributes and with the instrumental volatile composition. From the first four partial least squares factors 85% volatile compound variation explained 72% of descriptive sensory analysis data variation. Koppel *et al*. found lower percentages of data explained between descriptive sensory aroma attributes and volatile compounds data [13]. Better explanation of data variation may be caused by less variation within the dataset as well as using sensory flavor information instead of aromatics information to detect associations with instrumental volatile compounds concentration. This approach was considered more suitable as for both flavor evaluation and solid phase micro extraction (SPME) the sample is prepared in a similar manner: moisture is added first and next the sample is warmed to enhance aromatics extraction. 

Some associations were found among sensory flavor and aftertaste attributes and volatile compounds. Vitamin flavor (Vitfl) and vitamin aftertaste (Vitat) seemed to be related to limonene (A34), 4-methyl pentanoic acid (A32), and nonanal (A16). In addition associations were found among the attributes toasted (Toastfl), grain (Grainfl), stale (Stalefl), and oxidized oil flavor (Oxoilfl), oxidized oil (Oxoilat), metallic (Metat), liver (Liverat), fish (Fishat), and barnyard (Barnat) aftertaste, and volatiles 3-dodecene (A35, tentative identification), 2-butylfuran (A30), benzaldehyde (A11), 3-hydroxytoluene (A36), 2-nonanone (A23), 1-R-α-pinene (A33, tentative identification), and 2-pentylfuran (A31). Furthermore, stale aftertaste (Staleat) and fish flavor (Fishfl) were associated with volatiles hexanal (A5), 3-octen-2-one (A22), octen-3-ol (A1), 2-decen-1-ol (A2), and octanal (A12). This cluster of volatiles and sensory attributes are likely to indicate oxidation processes and rancid aromatics. Similar associations were found by Koppel *et al.* [13].

Baked samples (0B and 20B) were associated with volatiles methyl butyrate (A24), methyl octanoate (A26), methylpyrazine (A28), 3-furaldehyde (A6), and 2,5-dimethyl pyrazine (A29). Methylpyrazine may have popcorn-like, 2,5-dimethyl pyrazine roasted odors, 3-furaldehyde bread-like odors, and esters fruity odors [29]. The concentrations of these volatiles were small, grainy, and toasted, and brown flavor attributes were lower in intensity in baked than in extruded samples. This may address why the extruded samples were more related to these sensory characteristics. Similar associations were found among samples 20ME and 20LE and volatiles 2-octenal (A15), 2-heptenal(Z) (A10), 2-nonenal (A17), 2-hexenal(E) (A7), and methyl hexanoate (A25).

This study established a clear difference in sensory characteristics of baked and extruded pet foods not found in previous literature. Although this study has some limitations, such as the small number of samples included, there were sufficient unique characteristics between extruded and baked pet foods to show differences in sensory flavor and texture attributes. Texture is an important factor in determining pet food palatability. According to Gibson and Alavi, extruded pet foods differ from baked pet foods by the proportion of gelatinized starch, and this may be related to pet food texture properties [8]. Furthermore, according to a review by Houpt and Smith, dogs have a preference towards canned and semi-moist meats when compared to dry foods, but no palatability studies have compared baked and extruded pet foods [26]. Anecdotal evidence from the pet trade would suggest that differences among extruded kibbles could influence product acceptability. It would be of interest to determine whether cooking type (extrusion vs. baking) would influence dog food choice.

As an interim step to that question, a human sensory panel was used instead of a panel of animals for sensory evaluation in this study. In part because the animal panel, unfortunately, is not able to describe food aromatics, flavor sensations, or texture attributes, while a trained human panel can. Ideally, these two sensory evaluations would complement each other and so animal preference could be translated via humans into usable information for the industry and scientists. Furthermore, instrumental measurements capable of identifying volatile compounds and then relating this to animal preferences would be of great interest.

## 4. Conclusions

This study demonstrated differences in baked and extruded pet foods appearance, texture, and flavor characteristics. Baked samples were lighter in color and had lower levels of attributes that indicated rancidity (*i.e.*, fish flavor) than extruded pet foods. Extruded pet foods were more cohesive in mass, more fracturable, hard, and crisp, but less powdery than baked samples. Fresh meat inclusion tended to affect bitterness, fishiness, and cohesiveness of mass of pet foods regardless of processing method. High thermal to mechanical energy ratio during extrusion resulted in less musty and more porous kibbles. The main volatile compounds included aldehydes, such as hexanal and heptanal, ketones, and alcohols. Extruded samples did not contain any methylpyrazine, while baked samples did not contain any 2-butyl furan. Overall, extruded samples seemed to result in greater flavor intensity and aftertaste attributes in comparison to baked samples. Flavor, odor, and texture characteristics associations with palatability should be of interest to future studies.
